# Valorization of Various Natural Substrates as Alternative Carbon Sources in Fermentation Media for Pullulan Production by Domestic *Aureobasidium pullulans* AZ‑6

**DOI:** 10.1021/acsomega.5c08620

**Published:** 2026-01-23

**Authors:** Gamze Nur Mujdeci, Melek Tijen Bozdemir, Zekiye Yesim Ozbas

**Affiliations:** † Faculty of Engineering, Department of Food Engineering, 162313 Hitit University, 19030 Çorum, Turkey; ‡ Faculty of Engineering, Department of Food Engineering, 37515 Hacettepe University, 06800 Ankara, Turkey; § Faculty of Engineering, Department of Chemical Engineering, Hacettepe University, 06800 Ankara, Turkey

## Abstract

This study investigates the potential of various agro-industrial and food wastes as alternative carbon sources for cost-effective and sustainable pullulan production by a domestic, melanin-free strain of *Aureobasidium pullulans* AZ-6. Nine natural substrates, including cheese whey, molasses, grape pomace, sugar beet pulp, melon rind, watermelon rind, onion waste, carrot peels, and sweet potato, were pretreated and used in supplemented fermentation media. Batch fermentations were carried out under optimized conditions (pH 6.48; 24.2 °C; 100 strokes per minute), and biomass, extracellular polysaccharides (EPS), pullulan yields, and sugar consumption kinetics were evaluated. Among the tested substrates, molasses exhibited the highest EPS (17.12 g/L) and pullulan titers (14.55 g/L), as well as the greatest specific growth rate (0.104 h^–1^). Although cheese whey supported the highest biomass (13.03 g/L), its pullulan productivity was relatively low (4.98 g/L). Other substrates, such as sweet potato, onion waste, and carrot peels, also showed promising pullulan yields (8.24–8.70 g/L) and substrate utilization rates. This study evaluates the potential of several waste-derived substrates for pullulan biosynthesis and contributes to the circular bioeconomy by valorizing underutilized resources. To the best of our knowledge, sugar beet pulp, melon rind, watermelon rind, onion peel, and carrot peel have been investigated as substrates for pullulan production for the first time in the literature.

## Graphical abstract

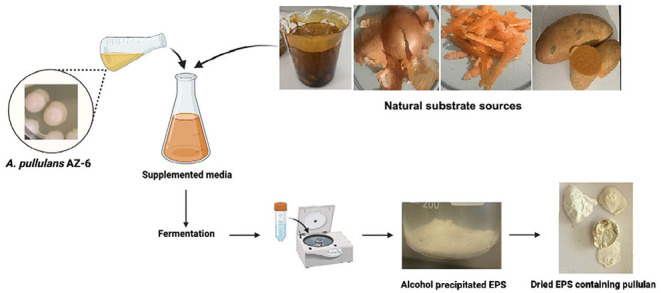

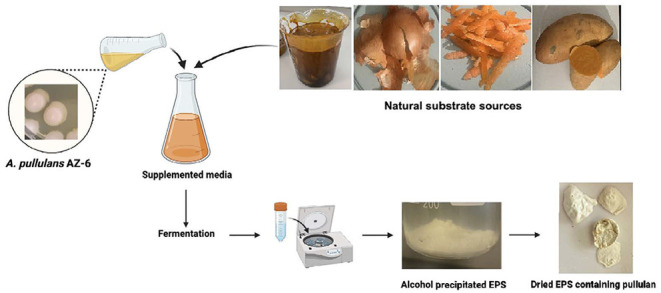

## 1 Introduction

Pullulan is a linear exopolysaccharide synthesized by various strains of the fungus *Aureobasidium pullulans*, composed of repeating maltotriose units connected through alternating α-(1 → 6) and α-(1 → 4) glycosidic bonds. First isolated by Bauer in 1938, its structural features were elucidated by Bernier in 1958, and it was formally named “pullulan” by Bender in 1959.^1^

Pullulan’s remarkable physicochemical attributes including high water solubility, edibility, biodegradability, and the absence of taste or odor combined with a nontoxic, noncarcinogenic, nonmutagenic, and nonimmunogenic safety profile, recognized as Generally Recognized as Safe (GRAS), have led to its widespread adoption across the food, pharmaceutical, and cosmetic industries.^2^ Furthermore, pullulan’s functional performance can be fine-tuned via blending with other polymers or through targeted chemical modifications, thereby expanding its applicability in specialty formulations.^3^

Despite these advantages, the cost of pullulan production estimated at approximately USD 25 per kg remains significantly higher than that of many other microbial polysaccharides, primarily due to the expense of sucrose (or other carbon sources), nitrogen, and supplementary nutrients in the fermentation medium.^4^ Media constituents alone can contribute up to 30% of total production costs, making the exploration of low-cost, renewable substrates a critical factor in improving economic feasibility.^4^

In line with circular economy principles, the valorization of agricultural and agro-industrial byproducts as alternative feedstocks enables cost-efficient and waste-minimizing pullulan production.^5^ Continuous research into such substrates not only supports environmental stewardship but also paves the way for more cost-effective commercial processes.

A variety of agro-based residues have been explored for this purpose. However, comparative kinetic analyses under identical conditions remain scarce. Some studies in which various agricultural wastes have been used for pullulan production are presented in Table 1. As seen in Table 1, several studies have demonstrated successful pullulan production using rice hull/bran,^6–8^ potato wastes,^9–11^ cassava wastes,^12–14^ sesame seed oil cake,^15^ and Asian palm kernel residues.^6,13^ Residues derived from the fruit processing industry such as apple pomace and banana peel have also been evaluated under solid-state and submerged fermentation conditions for pullulan production.^16,17^ In a recent study, sugar cane bagasse hemicellulosic hydrolysate was used as a carbon source for *A. pullulans* ATCC 42023, yielding up to 28.6 g/L ± 1.43 g/L pullulan in a bubble column reactor, highlighting the feasibility of lignocellulosic biorefineries for biopolymer production.^18^

**Table 1:** Some Natural Substrates That Have Been Used in Pullulan Fermentation

substrate	strain	refs
almond hull	*A. pullulans*	^5^
apple pomace	*A. pullulans* MTCC 1991	^16^
banana peel	*A. pullulan* MTCC 2195	^17^
brewery waste	*A. pullulans* P56	^18^
bakery waste	*A. pullulans* MTCC 2195	^19^
cashew apple juice		
cassava flour		
corn flour		
cassava starch	*A. pullulans* MTCC 1991	^20^
cassava bagasse	*A. pullulans* MTCC 2670	^13^
cassava waste	*A. pullulans* MTCC 1991	^14^
citrus peels	*A. pullulans* AZ-6	^21^
chestnut shells		
corn steep liquor	*A. pullulans* ATCC 42023, RBF 4A3, KY767024	^22–25^
corn syrup	*A. pullulans* NYS-1, NYSRP-1, ATCC 201253	^26,27^
coconut byproducts	*A. pullulans* MTCC 2195, MTCC 2670	^6,28^
cottonseed oil cake	*A. pullulans* RBF 4A3	^24^
date syrup	*A. pullulans*	^29^
demineralized whey	*A. pullulans* P56	^30^
deoiled jatropha seed cake	*A. pullulans* RBF 4A3	^24,31^
deoiled cashew nut seed cake	*A. pullulans* RBF 4A3	^23^
dried and fresh hazelnut husks	*A. pullulans* AZ-6	^21^
grape pulp/pomace	*A. pullulans* AZ-6, NRRLY 6220	^21,32^
hazelnut shells	*A. pullulans* AZ-6	^21^
huangjiu Lees	*A. pullulans* LL1	^33^
jackfruit seed	*A. pullulans* NCIM 1049, MTCC 2195	^34,35^
jerusalem artichoke root	*A. pullulans* Y-4137	^10^
molasses	*A. pullulans* P56, ATCC 42023, MTCC 2195, AZ-6, KY767024	^21,22,25,35–37^
mustard seed oil cake	*A. pullulans* RBF 4A3	^24,29^
olive oil mill waste	*A. pullulans* NRRLY 6220	^32^
palm sugar (Jaggery)	*A. pullulans* CFR-77, RBF 4A3, MTCC 2195	^23,38,39^
palm kernel	*A. pullulans* MTCC 2670	^6,40,41^
peat moss hydrolysate	*A. pullulans* 2552, 140B and 142	^42,43^
potato wastes	*A. pullulans* NRRLY 6220, P56, 201253, HIT-LCY3^T^, Y4137	^10,11,44–46^
residual brewery’s yeast	*A. pullulans* Y2092	^47^
rice wastes	*A. pullulans* ATCC 4202, CCTCC M 2012259, MTCC 2670, RBF 4A3	^6,7,22,24,29^
sesame seed oil cake	*A. pullulans* KY767024	^15^
soybean waste	*A. pullulans* NRRLY-6220, HP-2001, RBF 4A3, HP-2001	^24,29,48,49^
sweet potato	*A. pullulans* AP329, MTCC 2195	^50,51^
sugar cane bagasse	*A. pullulans* LB83, ATCC 42023	^18,52^
wheat straw	*A. pullulans* ATCC 42023, MTCC 2670	^6,22^

The present study evaluates a broad set of agro-industrial byproducts and food wastes, including cheese whey, molasses, grape pomace, sugar beet pulp, melon rind, watermelon rind, onion peel, carrot peel, and sweet potato, as alternative carbon sources for pullulan production by the indigenous, melanin-free strain *A. pullulans* AZ-6. Notably, sugar beet pulp, melon rind, watermelon rind, onion peel, and carrot peel are being tested for pullulan production for the first time in the literature, representing a significant advance in the valorization of underexplored waste streams. The aim of this study was to systematically compare the suitability of selected agro-industrial wastes as alternative carbon sources for pullulan production by *A. pullulans* AZ-6 under identical, previously optimized fermentation conditions.

## 2 Materials and Methods

### 2.1 Materials

#### 2.1.1 Microorganism

*Aureobasidium pullulans* AZ-6, previously isolated and taxonomically characterized from freshly harvested Gemlik olives in our laboratory, was used for experimental analyses.^53^ This strain was maintained on YM agar slants at 4 °C following inoculation into Yeast Extract Malt Extract (YM) broth and incubation at 28 °C for 48 h. For long-term preservation, the culture was stored in Yeast Extract Peptone Dextrose (YEPD) broth supplemented with 20% glycerol at −70 °C.

#### 2.1.2 Natural Substrates and Procurement

To assess alternative carbon sources for pullulan production, a variety of agro-industrial byproducts and food wastes collected from different regions of Turkey were utilized. These substrates included cheese whey, molasses, grape pomace, sugar beet pulp, melon rind, watermelon rind, onion waste, and carrot peels. Additionally, sweet potato was evaluated as a natural substrate source. Cheese whey was obtained from a local dairy facility (Dalgiclar Ciftliği), while molasses and sugar beet pulp were provided by the Safi Corum Sugar Factory in Corum province. Grape pomace was collected from Kavaklıdere Wines in Ankara. Sweet potatoes were sourced from a local farmer in Adana, and the remaining fruit and vegetable wastes were procured from local markets in Ankara.

### 2.2 Methods

#### 2.2.1 Inoculum Preparation

The inoculum of *A. pullulans* AZ-6 was prepared by cultivating the strain on YM agar slants at 28 °C for 48 h. Following activation, two loopfuls of the culture were transferred into 250 mL cotton-plugged Erlenmeyer flasks containing 50 mL of sterilized growth medium composed of (g/L): sucrose, 30; (NH_4_)_2_SO_4_, 2.0; yeast extract, 3.0; K_2_HPO_4_, 5.0; MgSO_4_·7H_2_O, 0.2; and NaCl, 1.0. The cultures were incubated at 28 °C in shaking water baths at 100 rpm for 48 h.^53^ These cultures were then used as an inoculum in subsequent fermentation experiments. Initial cell concentrations in the fermentation flasks were confirmed by spread plating on YM agar following serial dilutions in 0.85% NaCl solution.

#### 2.2.2 Pretreatment of Natural Substrates

Cheese whey, molasses, grape pomace, and sugar beet pulp were used as food industry byproducts, while melon rind, watermelon rind, onion waste, and carrot peels were selected as fruit and vegetable wastes. Sweet potato was also used as a natural substrate source in the study due to its rich complex carbohydrate content. Each substrate underwent a tailored pretreatment process based on its structural characteristics and was analyzed to determine initial sugar concentrations, considering the predominant sugar types and their expected quantities, as reported in the literature.

Cheese whey was used directly without any pretreatment. Molasses was diluted with distilled water in a 1:10 ratio to achieve a final sucrose concentration of 100 g/L.

Grape pomace was mixed with hot water at about 70 °C with a ratio of 3:1 (w/v), and the mixture was blended for 30 min and then filtered using a clean muslin cloth to obtain the extract to be used as a substrate for fermentation. The same procedure was also used for sugar beet pulp.^32^

In the case of melon, watermelon, and carrot peels and onion waste, the raw materials were first thoroughly washed. Melon and watermelon rinds were peeled to a thickness of approximately 2 cm, and carrot peels were peeled to about 0.1 cm. For onion samples, the outer brown peel, the thick white inner layer, and the top of the bulb were removed and used to prepare the fermentation medium in the experiments. Sugar extraction was performed using hot water extraction by adding hot water (∼70 °C) at a 3:1 (w/v) ratio. The peels were blended and intermittently stirred for 30 min, then filtered through a muslin cloth and centrifuged at 4100 rpm for 20 min.^32^

For sweet potatoes, tubers were washed, peeled, and chopped into uniform cubes (∼3 cm × 3 cm). Hot water (∼70 °C) was added at a 1:1 (w/v) ratio, and the mixture was blended and intermittently stirred for 30 min before being filtered through a muslin cloth.^32^

All extracts obtained, including molasses solution and cheese whey, were used supplemented with additional nutrients [(g/L): (NH_4_)_2_SO_4_, 2.0; peptone, 11.3; K_2_HPO_4_, 5.0; MgSO_4_ 7H_2_O, 0.2; and NaCl, 1.0] other than sucrose in the fermentation medium described in our previous optimization study.^53^ Prior to use in fermentation experiments, all media were sterilized in an autoclave at 121 °C for 15 min, and the initial pH values were adjusted to 6.48, which was previously determined as the optimum value.

#### 2.2.3 Equipment and Fermentation Conditions

Fermentations were carried out under standardized conditions in batch systems using shaking water baths [Grants SS40-D (U.K.), Haake SWB-20 (Germany), and Nuve ST-402 (Turkey)], which allowed for controlled temperature and agitation. The working volume was 150 mL in 300 mL Erlenmeyer flasks. Fermentation experiments were conducted under the previously optimized conditions (pH 6.48; 24.2 °C) as reported by Mujdeci et al.^53^ Throughout the experiments, the agitation rate was maintained at 100 strokes per minute. Samples were taken approximately every 24 h to determine biomass, extracellular polysaccharides (EPS), pullulan, and residual sugar concentrations. Maximum yields and kinetic parameters, including specific growth rate (μ), specific product formation rate (*q*), and substrate consumption rate (*r*_s_) were subsequently calculated.

#### 2.2.4 Analytic Assays

The sugar concentrations of the fermentation media were determined using analytical methods selected based on the dominant sugar types in each substrate. Lactose concentration in the cheese whey-based medium was quantified using the dinitrosalicylic acid (DNS) method.^54^ For molasses, the sucrose content was determined using the phenol-sulfuric acid method. Similarly, the initial sucrose concentrations in sweet potato and sugar beet pulp extracts were also quantified using the phenol-sulfuric acid method.^55^ In the case of melon, watermelon, and carrot peel extracts, initial concentrations of fructose and glucose were measured by using d-fructose/d-glucose enzymatic kits (Megazyme, Ireland). For onion waste extract, the d-glucose content was determined using the d-glucose HK enzymatic test kit (Megazyme, Ireland).

Microbial growth was monitored by determining biomass concentration, expressed as the dry cell weight (g/L). At approximately 24 h intervals, 10 mL of culture was sampled and centrifuged at 5000 rpm for 20 min. The resulting pellets were dried at 80 °C in a laboratory oven until a constant weight was achieved. Biomass concentration was expressed as g/L.

Extracellular polysaccharides (EPS) were quantified by ethanol precipitation. After centrifugation, culture supernatants were treated with two volumes of cold ethanol (99.8%) and kept at 4 °C (in a fridge) for 24 h to allow EPS precipitation. The resulting EPS was collected by centrifugation at 4100 rpm for 20 min, dried at 80 °C, and weighed. EPS concentration was expressed as g/L.

Pullulan content within the EPS fraction was specifically quantified using enzymatic hydrolysis with pullulanase (Promozyme D2, Sigma), following the method of Sharma et al.^24^ with slight modifications. EPS sample (20 mg/mL) was dissolved in distilled water, and 0.5 mL of the sample was mixed with 0.4 mL of phosphate-citrate buffer (pH 5.0) and 0.1 mL of pullulanase using a vortex mixer and then incubated at 40 °C for 2 h. The resulting maltotriose was quantified spectrophotometrically by using the DNS method at 540 nm. Pure pullulan (Merck) was used as the calibration standard. For determining the pullulan concentration in the analyzed EPS sample, the equivalent glucose concentration calculated for the EPS sample was ratioed to the equivalent glucose concentration obtained for pure pullulan. The resulting ratio was then multiplied by the concentration of EPS in the corresponding culture sample.

#### 2.2.5 Calculation of Fermentation Parameters

In this study, the maximum biomass, EPS, and pullulan concentrations, specific microbial growth rates (μ), maximum pullulan formation rates (*q*_p_), and substrate consumption rates (*r*_s_) were calculated based on the results of the fermentation experiments. The specific microbial growth rate during the exponential phase was determined from the semilogarithmic plot of dry biomass (*X*) versus time. The maximum pullulan formation rate was calculated according to eq 1:

1 $$q_{p}=\frac{1}{X}\frac{dC_{p}}{dt}$$

where *X* is the dry biomass of *A. pullulans* AZ-6, *C*_p_ is the pullulan concentration, and *t* is time. Substrate consumption rates (*r*_s_) were determined by calculating the slopes of the tangent lines at different time points from the curves showing changes in substrate concentrations over time.

## 3 Results and Discussion

Studies evaluating the production of pullulan and EPS in fermentation media obtained from various natural sources and supplemented with some synthetic components were carried out in two groups. While melon peel, grape pomace, watermelon peel extracts, molasses solution, and cheese whey were used as substrates in the first group, sweet potato, carrot peel, onion waste, and sugar beet pulp extracts were utilized in the second group of experiments. In both groups of experiments, the natural fermentation media were supplemented with several components, as described in Section 2.2.2.

Graphs showing the changes in EPS concentrations with time during fermentation in the first group of experiments are given in Figure 1. For these experiments, the initial inoculum concentration of the *A. pullulans* AZ-6 strain in the fermentation media was determined as 2.8 × 10^6^ CFU/mL.

**Figure 1:**
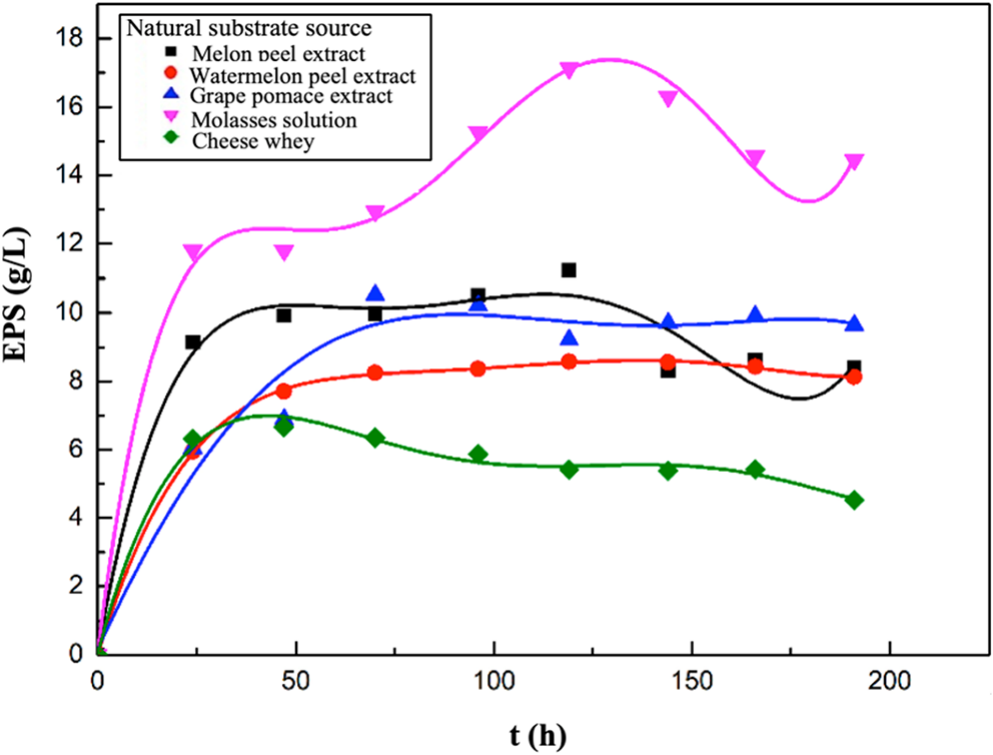
Variations in EPS concentrations with time for the media containing melon peel, watermelon peel, grape pomace extracts, molasses solution, or cheese whey.

In this study, the highest EPS concentration was obtained in the fermentation medium, where molasses was used as the substrate source. Subsequently, fermentation media used melon peel, grape pomace, watermelon peel extracts, and cheese whey as substrate sources, respectively. The maximum EPS concentrations achieved in the media containing molasses, melon peel, grape pomace, watermelon peel extracts, and cheese whey as carbon sources were determined to be 17.12, 11.22, 10.53, 8.58, and 6.66 g/L, respectively.

It was determined that the highest concentrations of EPS production occurred at the 120th hour of fermentation in the experiments where melon or watermelon peel extract or molasses was used as the natural substrate source; at the 73rd hour in the experiment where grape pomace was used as the substrate; and at the 49th hour in the experiment where cheese whey was used as the substrate.

Figure 2 presents graphs showing the changes of pullulan concentrations with time. In the experiments where melon peel extract, watermelon peel extract, or molasses solution were used as natural substrate sources, the highest pullulan concentrations were obtained at the 120th hour of fermentation, as 9.87, 8.32, and 14.55 g/L, respectively. In the experiments using grape pomace extract or cheese whey as natural sources, the highest pullulan concentrations were determined as 9.69 and 4.98 g/L, respectively, at the 73rd hour of fermentation.

**Figure 2:**
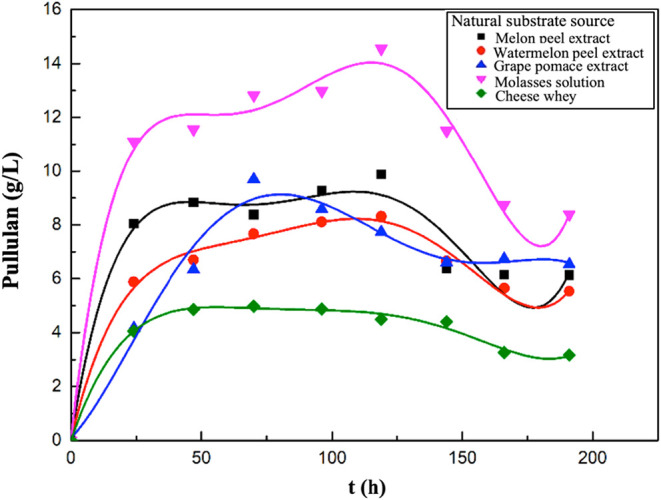
Variations in pullulan concentrations with time for the media containing melon peel, watermelon peel, grape pomace extracts, molasses solution, or cheese whey.

In these experiments, the changes in biomass concentrations over time are listed in Figure 3. In the supplemented fermentation media where molasses, cheese whey, grape pomace, and melon and watermelon peel extracts were used as natural substrate sources, the highest biomass concentrations were determined as 7.77, 13.03, 6.82, 3.95, and 5.36 g/L, respectively.

**Figure 3:**
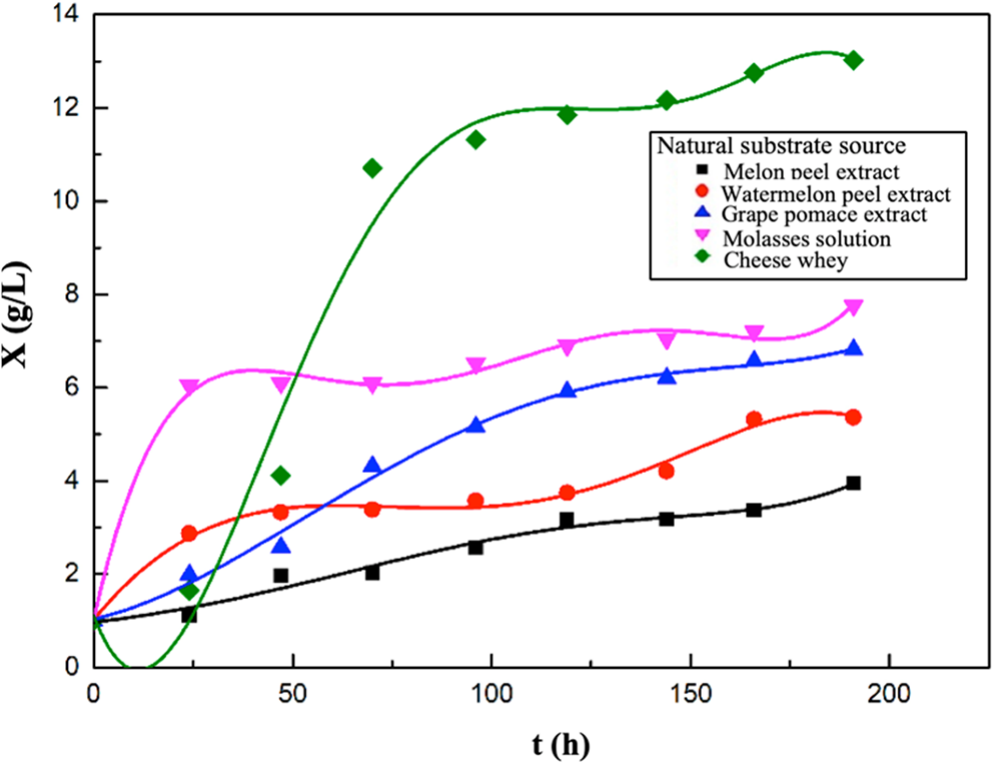
Variations in biomass concentrations with time for the media containing melon peel, watermelon peel, grape pomace extracts, molasses solution, or cheese whey.

The specific growth rates of the *A. pullulans* AZ-6 strain were also calculated for each fermentation medium used in these experiments. For this purpose, the ln *X* values were calculated from the graphs showing the changes in biomass concentrations over time in each experiment, and then graphs showing the changes in these values over time were plotted (Figure 4). The specific growth rates of the microorganism during the exponential growth phase were calculated accordingly. The specific growth rate (μ) of *A. pullulans* AZ-6 in fermentation media containing melon peel, watermelon peel, or grape pomace extracts as substrates was calculated as 0.023, 0.073, and 0.056 h^–1^, respectively. In media containing molasses solution or cheese whey, the specific growth rates were determined as 0.104 and 0.037 h^–1^, respectively.

**Figure 4:**
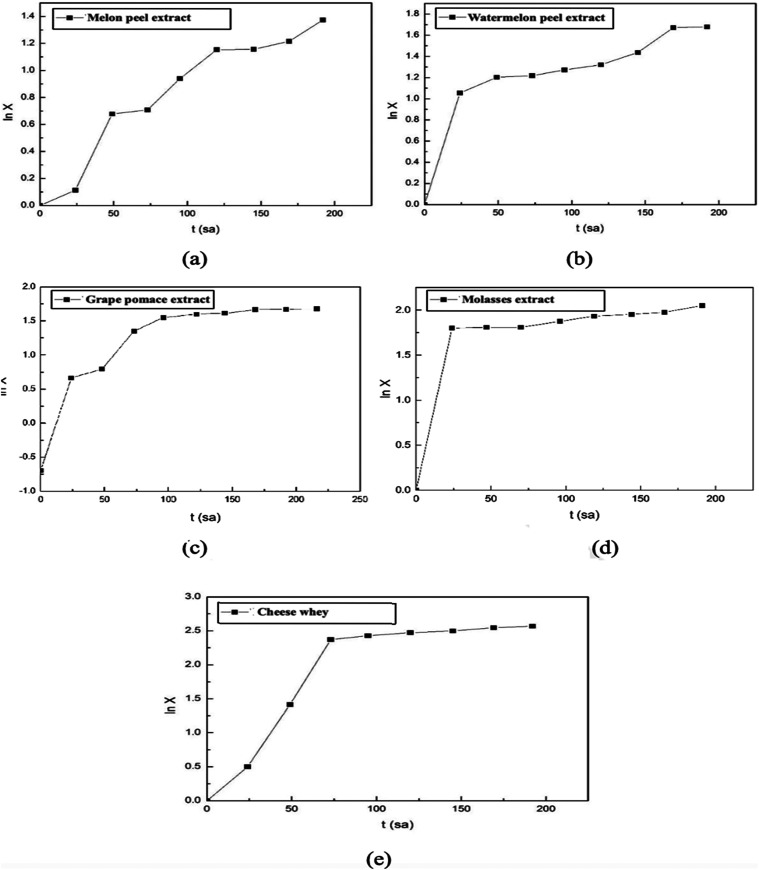
Variations in ln *X* values with time in the experiments conducted in fermentation media containing (a) melon peel extract, (b) watermelon peel extract, (c) grape pomace extract, (d) molasses solution, or (e) cheese whey as natural sources.

In this study, the highest specific product formation rates related to pullulan production (*v*_m_) were also calculated for these fermentation media. Specific pullulan production rates were calculated from the curves in Figure 2 representing the changes in pullulan concentrations with time by determination of the slopes of the tangent lines of the curve corresponding at a definite time and then dividing the slopes by the corresponding biomass concentrations obtained from Figure 3 at the same time. The highest *v*_m_ values were found to be 0.094, 0.122, and 0.115 g of pullulan/(g of biomass·h) for melon peel, watermelon peel, and grape pomace extract-based media, respectively, and 0.176 and 0.211 g of pullulan/(g of biomass·h) for media with molasses and cheese whey, respectively.

At this stage of the study, the concentrations of different types of sugars present in the supplemented fermentation media containing various natural sources were also determined.

The graphs showing the changes in glucose and fructose concentrations with time in the fermentation medium where melon peel extract, watermelon peel extract, and grape pomace extract were used as substrates are presented in Figure 5.

**Figure 5:**
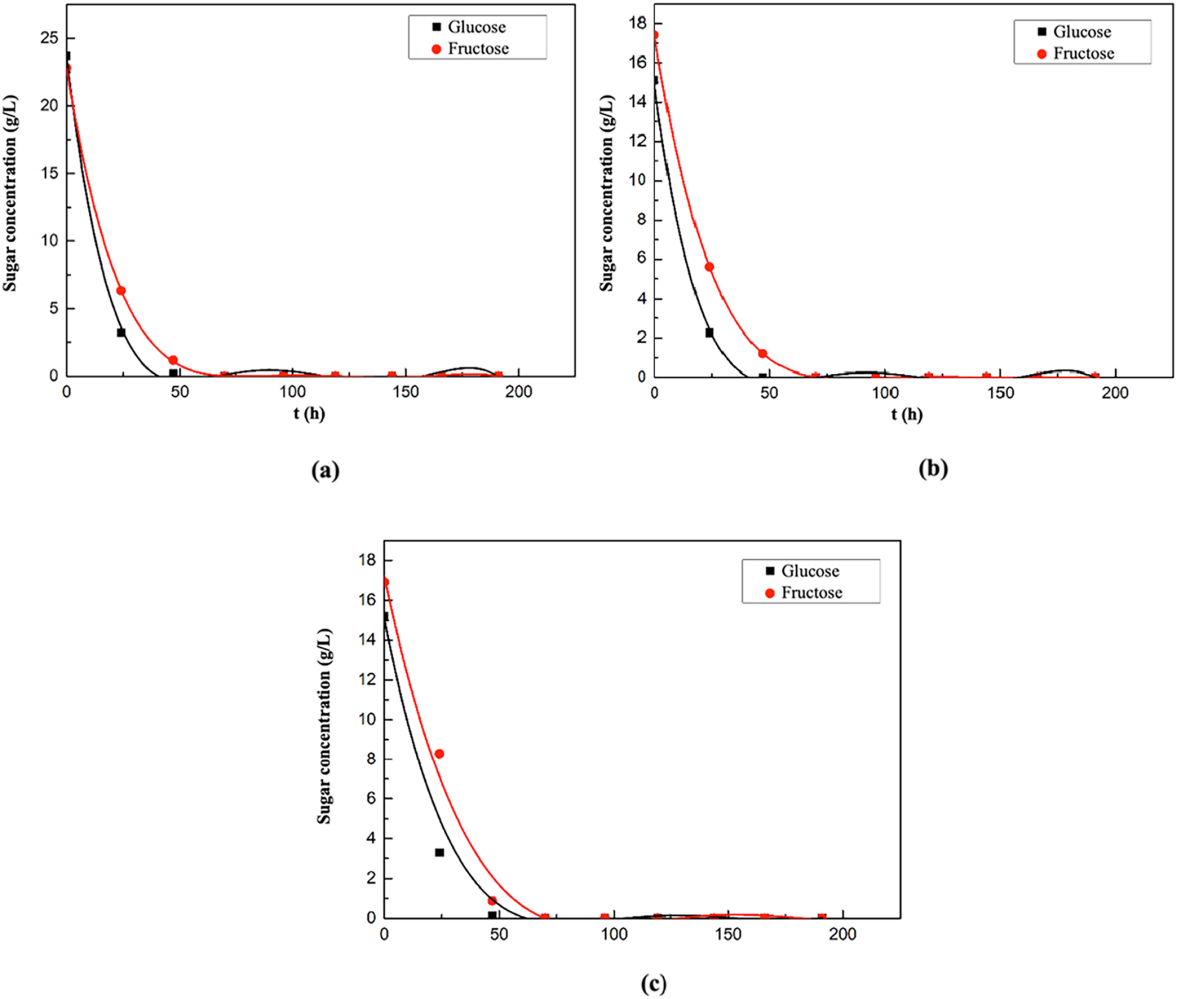
Changes in glucose and fructose concentrations with time in the supplemented fermentation media containing melon peel extract (a), watermelon peel extract (b), and grape pomace extract (c) as natural substrates.

It was observed that both glucose and fructose in the fermentation medium containing melon peel extract were completely consumed by the 70th hour of fermentation (Figure 5a). As shown in Figure 5b, glucose and fructose in the watermelon peel extract-based medium were completely consumed by the 40th hour and 70th hour of fermentation, respectively. In the fermentation medium containing grape pomace extract, both glucose and fructose were completely consumed by the 70th hour of fermentation (Figure 5c).

The graphs showing the changes in glucose and fructose consumption rates with time in the fermentation medium where melon peel extract, watermelon peel extract, and grape pomace extract were used as the substrate are provided in Figure 6. In the medium containing melon peel extract, the highest consumption rates for glucose and fructose were observed at the onset of fermentation, measured as 0.85 g of glucose/(L·h) and 0.69 g of fructose/(L·h), respectively (Figure 6a). Similarly, in the watermelon peel extract-based medium, the maxima of the sugar consumption rates were also recorded at the beginning of fermentation, corresponding to 0.54 g of glucose/(L·h) and 0.49 g of fructose/(L·h), respectively (Figure 6b). In the medium supplemented with grape pomace extract, the highest glucose and fructose consumption rates were again detected at the start of fermentation, calculated as 0.50 g of glucose/(L·h) and 0.36 g of fructose/(L·h), respectively (Figure 6c).

**Figure 6:**
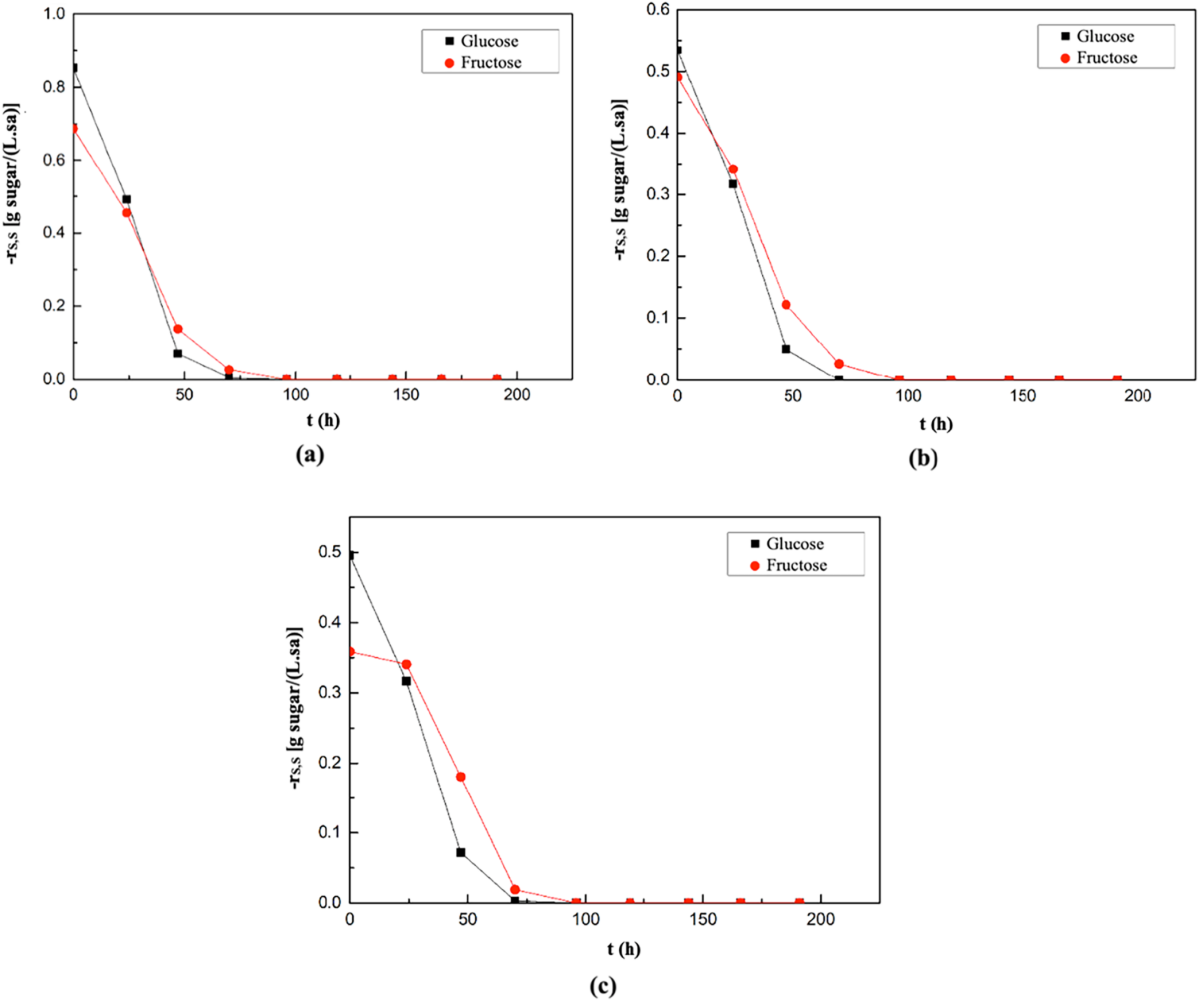
Changes in glucose and fructose consumption rates with time in the supplemented fermentation media containing melon peel extract (a), watermelon peel extract (b), and grape pomace extract (c) as natural substrates.

In the experiment where molasses was used as the natural substrate, the changes in the sucrose concentration of the medium and the consumption rate of sucrose by *A. pullulans* AZ-6 with time are shown in Figure 7a,b, respectively. In this fermentation medium, sucrose was completely consumed by the 169th hour of fermentation. Moreover, the maximum sucrose consumption rate in this medium was determined as 1.65 g of sucrose/(L·h) at the beginning of fermentation (Figure 7b).

**Figure 7:**
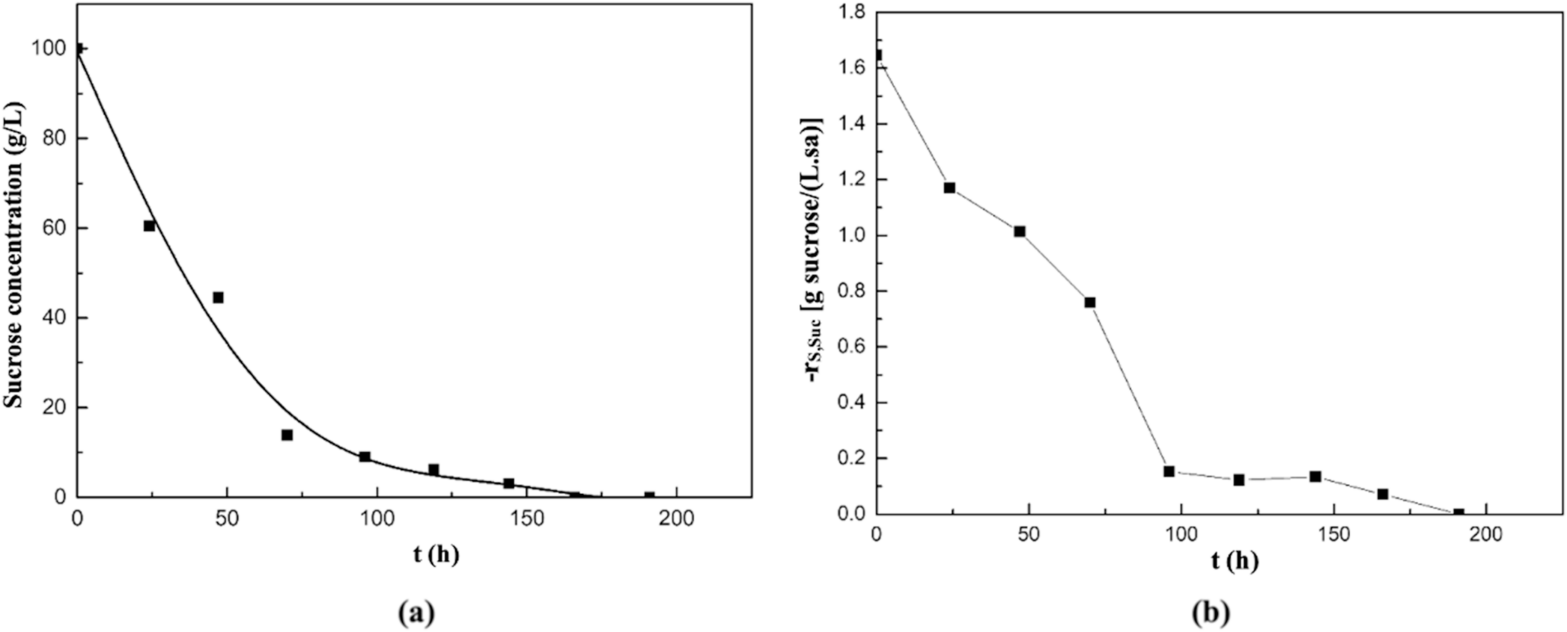
Changes in sucrose concentration (a) and its consumption rate (b) with time in the supplemented fermentation medium containing molasses solution as the natural substrate.

The graph showing the changes in lactose concentration in the fermentation medium with time, where cheese whey was used as the substrate, is presented in Figure 8a, and the graph depicting the lactose consumption rate of the strain during the fermentation is shown in Figure 8b. According to Figure 8a, lactose was completely consumed by 145th h of fermentation. As can be seen in Figure 8b, the maximum lactose consumption rate in this medium was determined as 0.38 g of lactose/(L·h), at the beginning of fermentation.

**Figure 8:**
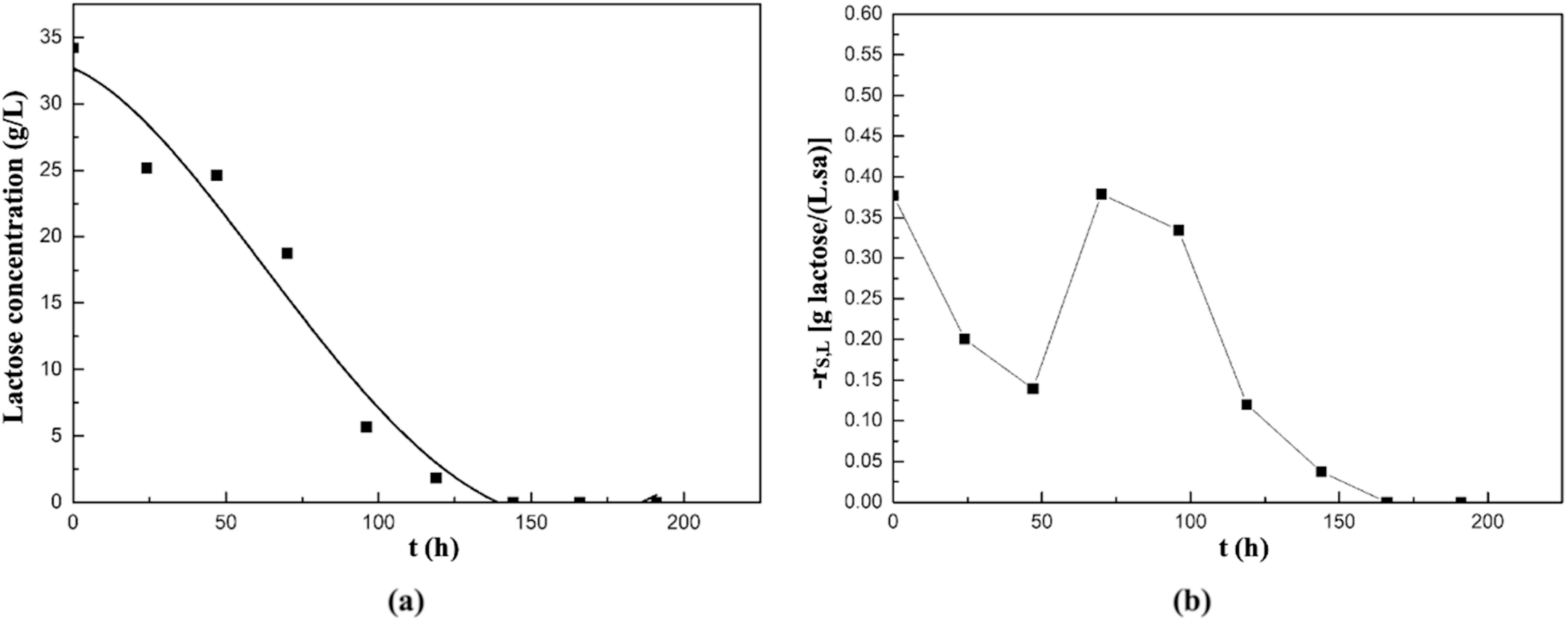
Changes in lactose concentration (a) and its consumption rate (b) with time in the supplemented fermentation medium containing cheese whey as the natural substrate.

In the second group of experiments conducted in this study, sweet potato, sugar beet pulp, onion waste, and carrot peel extracts were used as carbon source alternatives in supplemented fermentation media. Graphs showing the changes in EPS concentrations with time during fermentation in these experiments are given in Figure 9. In this study, the highest EPS concentration was obtained in the experiment, where sweet potato extract was used as the natural substrate in the fermentation medium. This was followed by experiments using carrot peel, onion waste, and sugar beet pulp extracts as natural substrate sources, respectively. The maximum EPS concentrations obtained in the fermentation media containing sweet potato, carrot peel, onion waste, and sugar beet pulp extracts as natural substrates were determined as 10.63, 9.43, 8.70, and 6.34 g/L, respectively. It was also observed that the highest EPS production occurred at the 96th hour of fermentation in media containing sugar beet pulp or onion waste extract, and at the 119th hour in media containing sweet potato or carrot peel extract.

**Figure 9:**
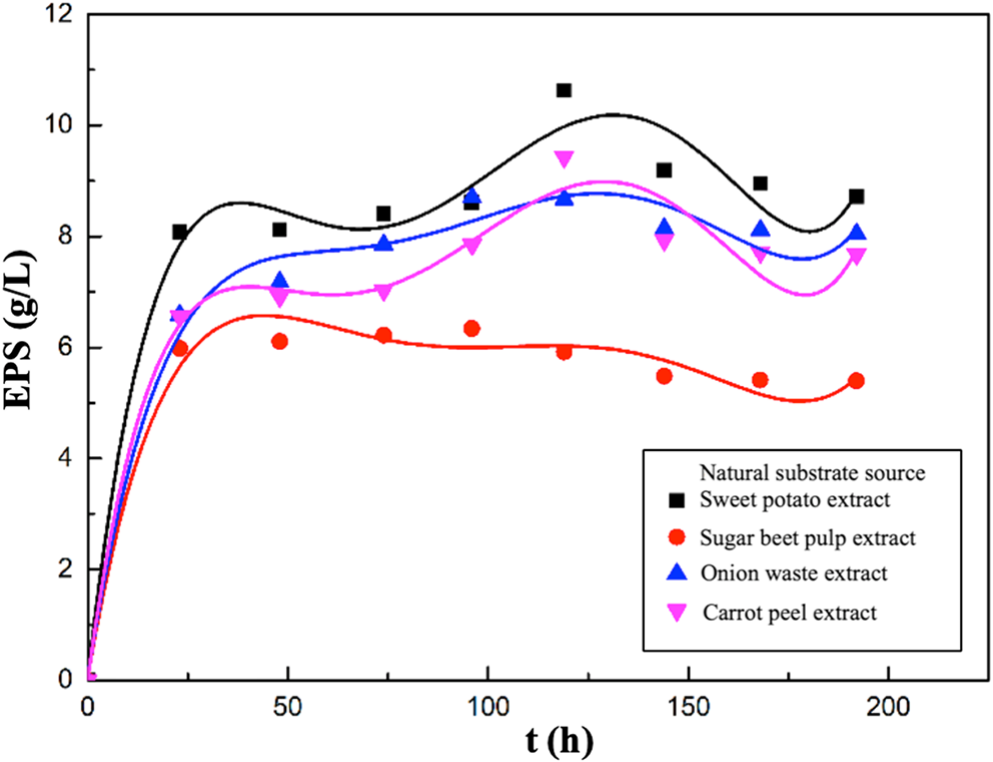
Variations in EPS concentrations with time for the media containing sweet potato, sugar beet pulp, onion waste, or carrot peel extract.

In the second group of experiments, graphs showing the changes in pullulan concentrations with time are presented in Figure 10. In the experiments where sugar beet pulp extract and onion waste extract were used as natural substrate sources, the maxima of the pullulan concentrations were determined as 6.28 and 8.18 g/L, respectively, at the 96th hour of fermentation. The highest pullulan concentration was measured as 8.24 g/L at the 74th hour of fermentation in the experiment where sweet potato extract was used as the substrate, and as 7.36 g/L at the 119th hour in the experiment where carrot peel extract was used as the natural substrate.

**Figure 10:**
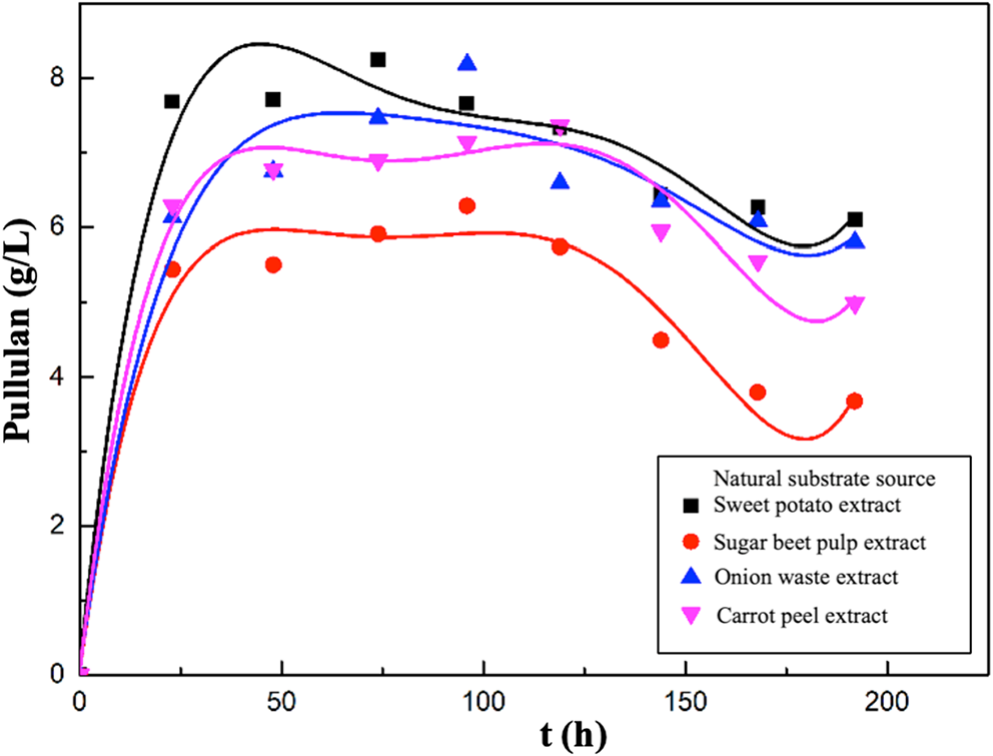
Variations in pullulan concentrations with time for the media containing sweet potato, sugar beet pulp, onion waste, or carrot peel extract.

The graphs showing the changes in biomass concentrations with time for the second group of runs are presented in Figure 11. In the experiment where sweet potato extract was used as the natural substrate source, the highest biomass concentration was determined as 4.37 g/L at the 116th hour of fermentation. The highest biomass concentrations were obtained as 2.05 and 5.11 g/L at the 192nd hour of fermentation, respectively, in the runs using onion waste and carrot peel extracts as natural substrates, while it was determined as 4.45 g/L at the 168th hour of fermentation in the sugar beet pulp extract-based medium.

**Figure 11:**
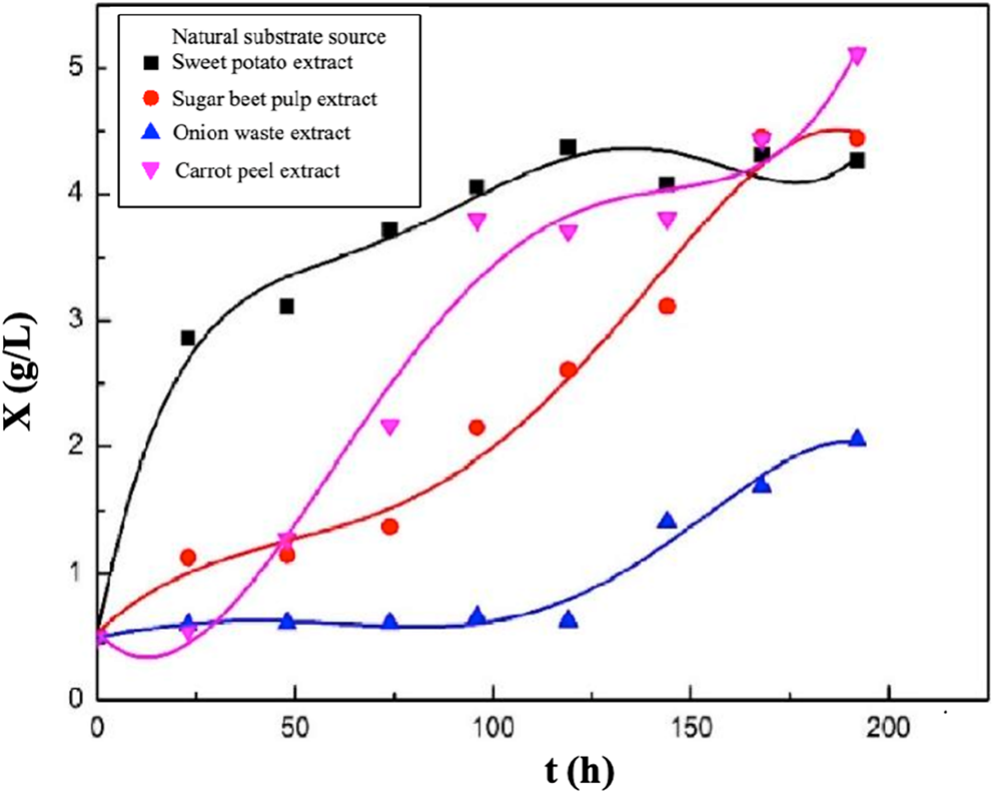
Variations in biomass concentrations with time for the media containing sweet potato, sugar beet pulp, onion waste, or carrot peel extract.

The specific growth rates of the *A. pullulans* AZ-6 strain were also calculated for the second group of the experiments. For this purpose, ln *X* values were calculated from the graphs showing the changes in biomass concentrations with time for each experiment, and the graphs showing the changes in ln *X* values with time were plotted (Figure 12) and then the specific growth rates of the microorganism were determined. The specific growth rates (μ) of *A. pullulans* AZ-6 in media containing sweet potato, sugar beet pulp, onion waste, and carrot peel extracts were calculated as 0.076, 0.035, 0.032, and 0.033 h^–1^, respectively.

**Figure 12:**
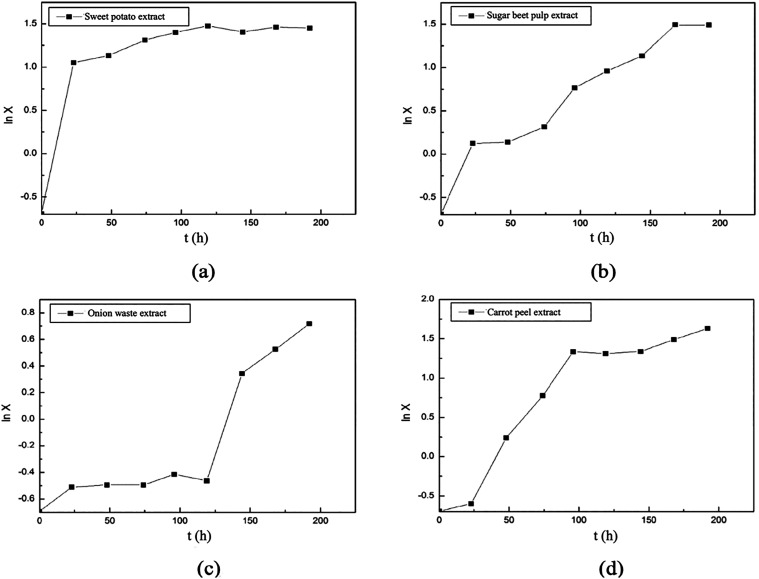
Variations in ln *X* values with time in the experiments conducted in fermentation media containing (a) sweet potato extract, (b) sugar beet pulp extract, (c) onion waste extract, and (d) carrot peel extract.

The maxima of the specific product formation rates for these runs were also calculated. The maximum specific product formation rates (*v*_m_) in the fermentation media containing sweet potato, sugar beet pulp, onion waste, and carrot peel extracts as natural substrates were determined as 0.117, 0.209, 0.444, and 0.497 g of pullulan/(g biomass·h), respectively.

Changes in sugar concentrations in the fermentation media with time were also determined in these experiments. In the medium containing sweet potato extract, changes in glucose, fructose, and total sugar (as sucrose) concentrations with time are shown in Figure 13a, and the changes in the sugar consumption rates are also shown in Figure 13b.

**Figure 13:**
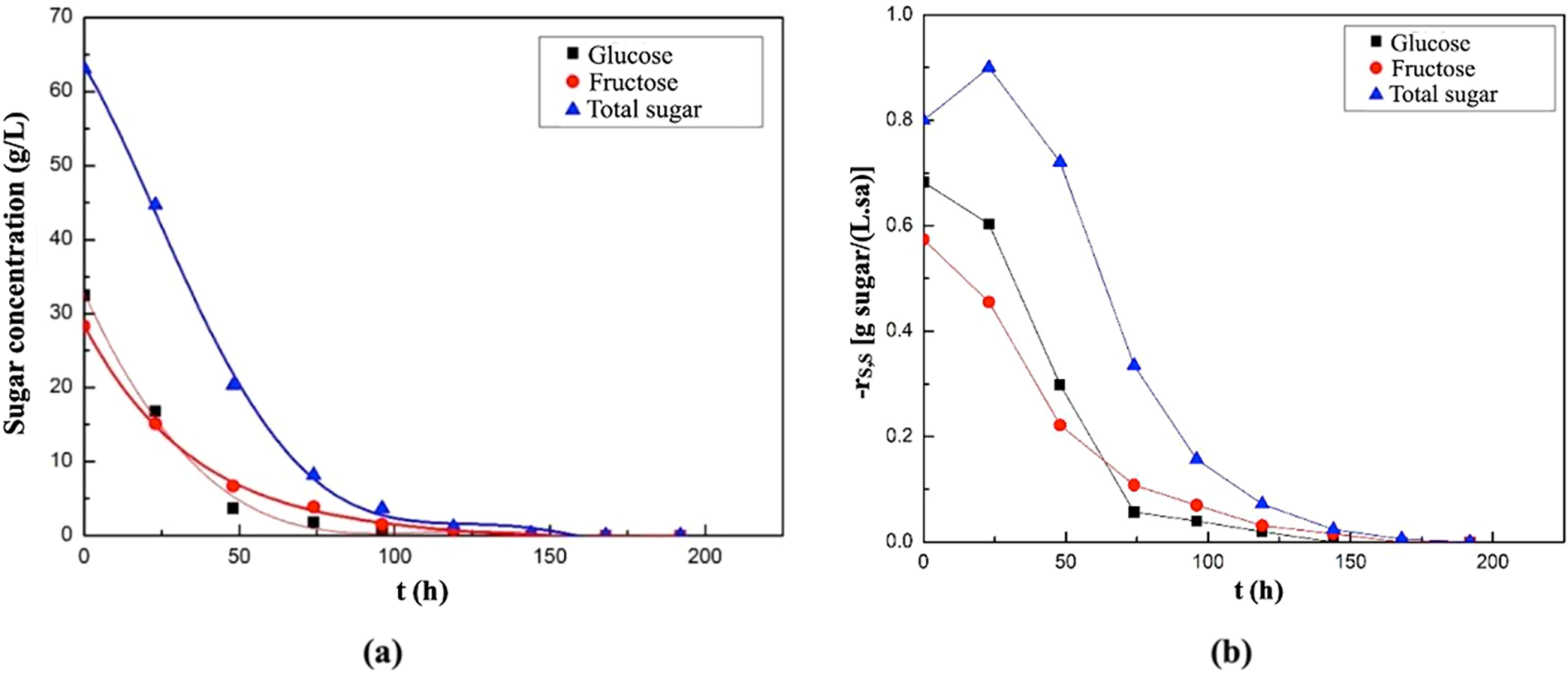
Changes in glucose, fructose, and total sugar concentrations (a) and their consumption rates (b) with time in the supplemented fermentation medium containing sweet potato extract as a natural substrate source.

According to the results in Figure 13a, glucose, fructose, and total sugar in the medium were completely consumed at the 119th, 144th, and 168th hours of fermentation, respectively. The highest glucose and fructose consumption rates were calculated as 0.68 g of glucose/(L·h) and 0.57 g of fructose/(L·h), respectively, at the beginning of the fermentation (Figure 13b). The highest total sugar consumption rate was found as 0.9 g sucrose/(L·h) and obtained at the 23rd hour of fermentation.

In the fermentation medium, where carrot peel extract was used as the natural substrate, the changes in glucose and fructose concentrations with time are shown in Figure 14a, and the corresponding consumption rates are shown in Figure 14b. In this experiment, *A. pullulans* AZ-6 consumed all of the fructose by the 119th hour and all of the glucose by the 144th hour. The highest consumption rates of glucose and fructose in this medium were calculated as 0.43 g/(L·h) and 0.41 g/(L·h), respectively, at the beginning of fermentation, and then both gradually decreased with time.

**Figure 14:**
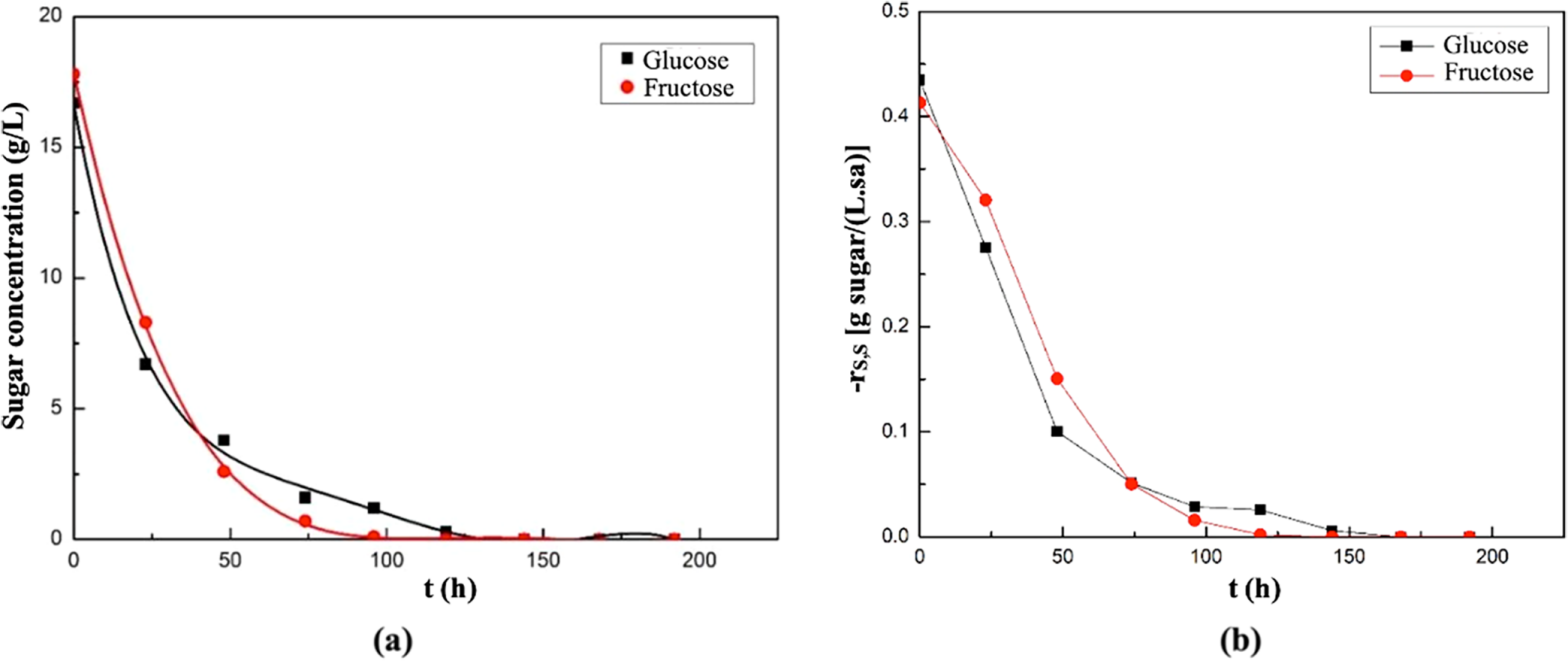
Changes in glucose and fructose concentrations (a) and their consumption rates (b) with time in the supplemented fermentation medium containing carrot peel extract as the natural substrate source

In the experiment using onion waste extract as the natural substrate, changes in glucose concentration with time are shown in Figure 15a. Additionally, the changes in glucose consumption rate with time are shown in Figure 15b. In this experiment, the glucose was completely consumed by the 144th hour, and the highest glucose consumption rate of the biomass was recorded as 0.87 g/(L·h) at the 23rd hour, and it was decreased sharply with time.

**Figure 15:**
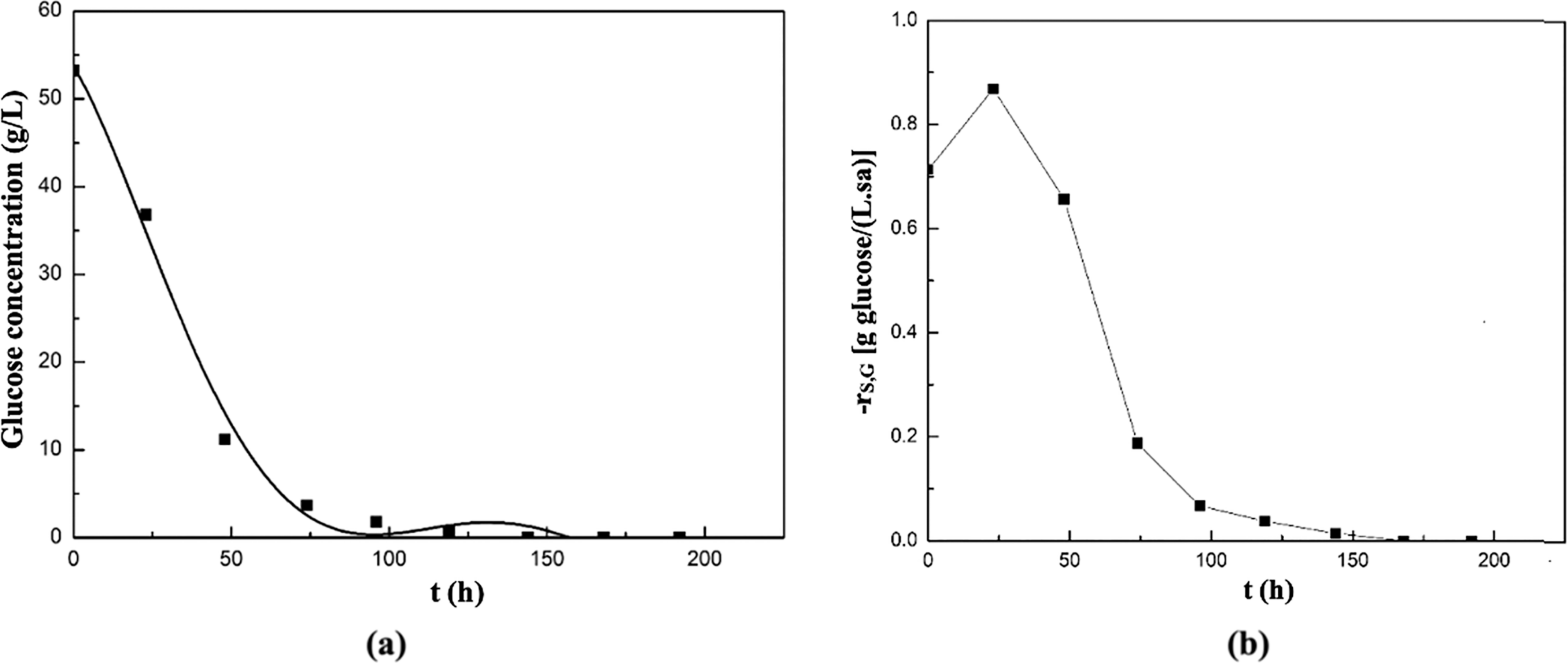
Changes in glucose concentration (a) and its consumption rate (b) with time in the supplemented fermentation medium containing onion waste extract as the natural substrate source.

In the fermentation medium containing sugar beet pulp extract, changes in sucrose concentration and its consumption rate are shown in Figure 16a,16b, respectively. In this experiment, sucrose was fully consumed by the 119th hour. The highest sucrose consumption rate, 0.52 g/(L·h), was obtained at the beginning of the fermentation and then it was observed to decrease exponentially with time.

**Figure 16:**
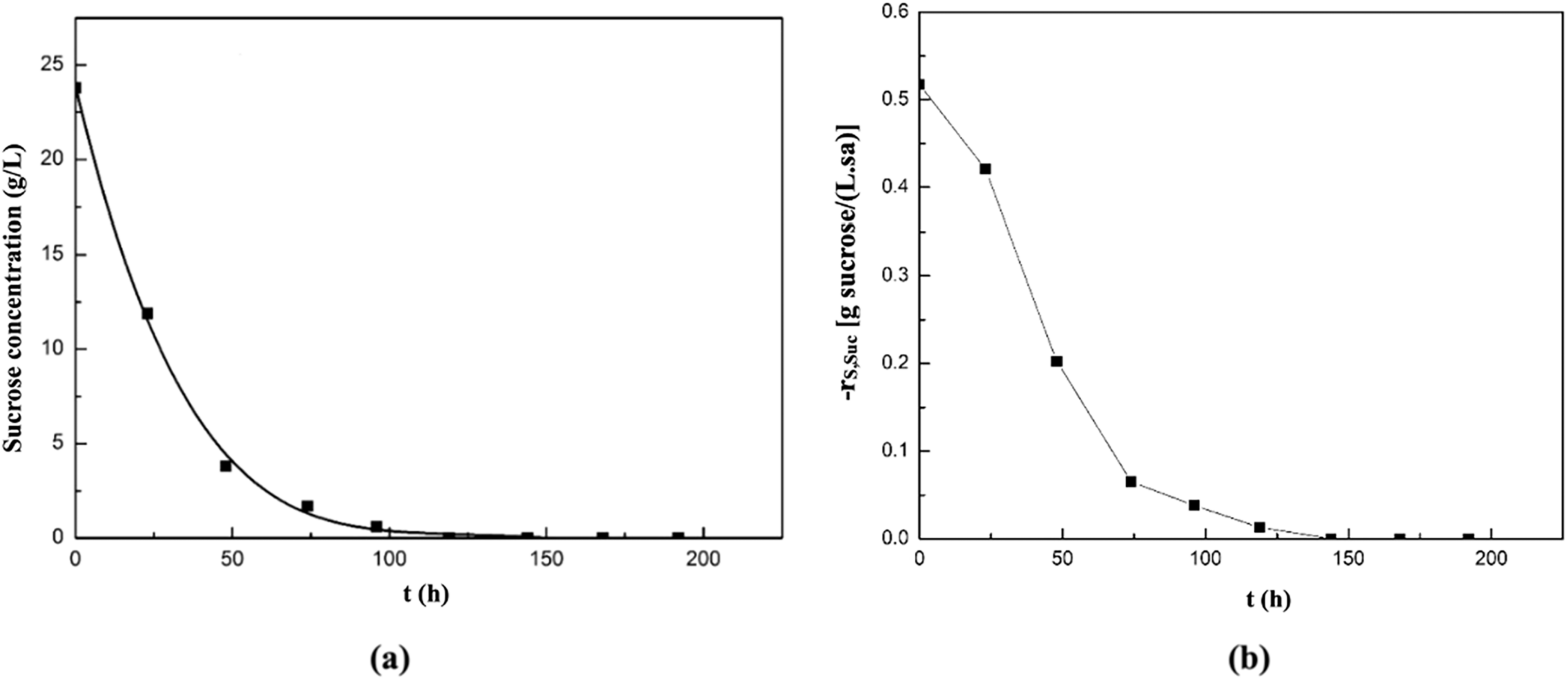
Changes in sucrose concentration (a) and its consumption rate (b) with time in the supplemented fermentation medium containing sugar beet pulp extract as the natural substrate source.

The maximum EPS, pullulan, biomass, and specific pullulan production rates and specific growth rate values determined in fermentation media where various natural substrates were used as alternative carbon sources are presented in Table 2.

**Table 2:** Effects of Change in Natural Substrate Type in Fermentation Medium Composition on the Maxima of EPS, Pullulan, Biomass, and Specific Pullulan Production Rates and Specific Growth Rates

natural substrate source	maximum EPS concentration (g/L)	maximum pullulan concentration (g/L)	maximum biomass concentration (g/L)	maximum specific pullulan production rate [(g pullulan/g mo·h)]	specific growth rate (1/h)
melon peel extract	11.22	9.87	3.95	0.094	0.023
watermelon peel extract	8.58	8.32	5.36	0.122	0.073
grape pomace extract	10.53	9.69	6.82	0.115	0.056
molasses solution	17.12	14.55	7.77	0.176	0.104
whey	6.66	4.98	13.03	0.211	0.037
sweet potato extract	10.63	8.24	4.37	0.117	0.076
sugar beet pulp extract	6.34	6.28	4.45	0.209	0.035
onion peel extract	8.70	8.18	2.05	0.444	0.032
carrot peel extract	9.43	7.36	5.11	0.497	0.033

Agro-industrial wastes represent nutrient-rich, low-cost substrates, whose valorization is both ecologically sound and economically advantageous. In this context, our comparative evaluation of nine agro-industrial byproducts for pullulan production by *A. pullulans* AZ-6 aligns with broader efforts to replace refined sugars with waste-derived carbon sources.^21,25^

Among the substrates tested, molasses solution exhibited the highest EPS concentration (17.12 g/L) and pullulan titer (14.55 g/L), as well as the greatest specific growth rate (0.104 h^–1^). These findings corroborate Oktay et al.,^21^ who reported a maximum pullulan concentration of 33.59 g/L using sugar cane molasses residue without auxiliary nutrients, and Zarei et al.,^25^ who achieved an 18.29 g/L yield under optimized molasses-corn steep liquor conditions. The superior performance of molasses likely reflects its high sugar content and mineral profile, which support both growth and polymer synthesis.

In contrast, cheese whey, although supporting the highest biomass accumulation (13.03 g/L), yielded relatively low pullulan (4.98 g/L) and EPS (6.66 g/L) concentrations, indicating that lactose-based substrates may preferentially drive cell proliferation rather than polysaccharide synthesis under the present conditions. This decoupling of growth and product formation suggests that whey-based media might require supplementation or process control. Roukas^30^ demonstrated that when lactose was enzymatically hydrolyzed, pullulan production increased markedly: a maximum polysaccharide concentration of 11.0 ± 0.5 g/L, biomass of 10.5 ± 0.4 g L^–1^, yield of 47.2 ± 1.8% and 93.2 ± 2.8% sugar utilization were achieved using whey (pH 6.5) with 25 g L^–1^ lactose, supplemented with K_2_HPO_4_ (0.5%), l-glutamic acid (1%), olive oil (2.5%), and Tween 80 (0.5%). Under these conditions, pullulan made up 40% of the crude polysaccharide fraction.^30^

The agro-industrial peels and pomaces displayed intermediate performances. Grape pomace extract supported moderate biomass (6.82 g/L) and pullulan production (9.69 g/L), whereas sweet potato and sugar beet pulp extracts yielded lower EPS and pullulan titers. Melon and watermelon rind extracts achieved respectable EPS levels (11.22 and 8.58 g/L, respectively) but differed in biomass formation, reflecting substrate compositional variability. Such outcomes may underline the need for tailored pretreatment or enzymatic hydrolysis protocols to enhance sugar availability from lignocellulosic residues.^3^

Notably, onion and carrot peel extracts, despite generating the low biomass (2.05 and 5.11 g/L, respectively), exhibited the highest specific pullulan formation rates [0.444 and 0.497 g pullulan/(g biomass·h)]. This elevated “productivity per cell” implies that under carbon-limited or stress-imposed conditions, *A. pullulans* reallocates resources preferentially toward pullulan synthesis rather than growth. Such substrates might therefore serve as valuable model systems for dissecting the regulatory mechanisms of pullulan biosynthesis and for developing high-yielding, low-biomass processes.

## 4 Economic and Environmental Outlook

The utilization of agricultural residues as natural substrate sources in pullulan fermentation offers notable economic and environmental advantages aligned with circular economy principles. Many of the substrates (melon peel, watermelon rind, grape pomace, onion peel, carrot peel, sugar beet pulp, sweet potato, whey, and molasses) evaluated in this study are generated in large quantities as low-value byproducts of the food processing sector. These residues typically have minimal or no commercial value and may even impose disposal costs on producers. Incorporating them into pullulan production therefore enables the valorization of waste streams while simultaneously reducing the reliance on refined sugars, which represent a major cost component in conventional fermentation media.

Although a full techno-economic analysis (TEA) was beyond the scope of the current work, preliminary observations suggest that replacing commercial sucrose with waste-derived substrates could significantly lower the substrate cost per unit of pullulan produced. Considering that carbon sources often account for a substantial fraction of the total operational expenditure in microbial biopolymer production, the use of these residues has the potential to reduce overall production costs and enhance process feasibility, particularly at an industrial scale.

From an environmental perspective, the diversion of organic wastes from landfill or uncontrolled decomposition contributes to reduced greenhouse gas emissions, while converting these byproducts into a value-added biopolymer supports resource efficiency and waste minimization. Moreover, as pullulan is a biodegradable and nontoxic polymer, its production using food waste-derived substrates aligns strongly with sustainability goals aimed at reducing environmental impact throughout the product life cycle.

## 5 Conclusions

This work highlights the feasibility of valorization of diverse agro-industrial residues as feedstocks for pullulan synthesis by the native, melanin-free strain *A. pullulans* AZ-6. In the study, the usability of these residues as carbon source alternatives in the fermentation medium used in pullulan production was evaluated. Sugar beet molasses stood out as the most effective single substrate, delivering the greatest polymer yield and productivity. In contrast, unmodified cheese whey favored cell proliferation over polysaccharide accumulation. The successful use of sugar beet pulp, melon rind, watermelon rind, onion waste, carrot peels, and sweet potato marks a novel contribution to the literature, underscoring the originality of this approach. While the findings of this study demonstrate the potential of agricultural residues as effective natural substrates for pullulan production, several challenges must be considered when transitioning from laboratory-scale fermentations to bioreactor-scale operations. High viscosity cultures, which are characteristic of pullulan producing systems, can significantly hinder mixing efficiency and reduce oxygen transfer rates, both of which are critical for maintaining optimal microbial growth and product formation.

Our future studies will focus on (i) cosubstrate strategies combining high sugar with nutrient-rich wastes/byproducts to balance biomass growth and pullulan synthesis, (ii) enzymatic or chemical hydrolysis to enhance monomer availability from lignocellulosic matrices, (iii) bioreactor-level process control to maximize volumetric productivity, and (iv) TEA and life cycle assessment (LCA).

## Data Availability

The data are available from the corresponding author upon reasonable request.
